# Supplementary material for: Transcriptomic and Metabolic Profiling Reveals a Lignin Metabolism Network Involved in Mesocotyl Elongation during Maize Seed Germination
Source: Plants (Basel). 2022 Apr 11;11(8):1034. doi: 10.3390/plants11081034 (PMC9027596; doi:10.3390/plants11081034)
Supplement: Supplementary file 1 [file plants-11-01034-s001.zip › plants-1646395-supplementary.pdf]

Table S1. Summary of quality preprocessing in N192 and Ji853 mesocotyls under 3 cm sowing depth (CK), 20 cm sowing depth (DSS), 4.16×10^{-3} M 24-epibrassinolide (EBR) induction at 20 cm sowing depth (DSS+EBR) conditions by RNA-sequencing (RNA-Seq) data.

Sample	Raw Date Size (bp)	Clean Date Size (bp)	Clean Date Rate (%)	Total Reads	Q20 percentage (%)	Q30 percentage (%)	GC (%)	Mapped Reads	Mapping Rate (%)
N192(CK)	8041438907	7453056733	92.68	48818227	98.04	93.18	53.83	43377315	88.85
N192(DSS)	8385320530	7484737105	89.26	50458344	98.11	94.02	53.27	45568930	90.30
N192(DSS+EBR)	8135207165	7951351483	97.74	51659460	97.99	94.65	54.15	45444827	87.97
Ji853(CK)	7945756549	7198855434	90.61	49974282	98.09	94.52	53.98	44946869	89.94
Ji853(DSS)	8402899726	7881919943	93.85	53956450	98.14	94.64	54.36	48566200	90.01
Ji853(DSS+EBR)	8563313670	8272161005	96.59	53691625	98.2	94.27	54.22	48462060	90.26
Average	8245656091	7707013617	93.46	51426398	98.10	94.21	53.97	46061033	89.56

Table S2. The quantitative real-time (qRT-PCR) primer sequence.

Gene name	Gene ID	Forward primer (5' to 3')	Reverse primer (5' to 3')	Chromosome	Gene Position (bp)
Actin1	*Zm00001d010159*	CGATTGAGCATGGCATTGTCA	CCCACTAGCGTACAACGAA	8	102413768..102417536
PAL	*Zm00001d003015*	CTCATCCGATTCCTCAATGC	GTCACGTTGGTGTTGAGCAG	2	29467845..29470586
PAL	*Zm00001d017276*	CCGAGTACCGCCAACCCT	CCAGTCGCTGCTTGCCTT	5	191474509..191476978
C4H	*Zm00001d016471*	CAACTACGGCGACTTCATCC	CGTTGATGTTCTCGACGATGT	5	163692318..163694217
HCT	*Zm00001d039947*	CGCATGCAGCCTGAAGAGAT	AACGAGGTCCAGCGGAGAGA	3	20216146..20226938
C3'H	*Zm00001d043174*	GGGAAGCCGTTGGTTGTAA	GGACGCACCGATCTTGATG	3	190625724..190628380
CCoAOMT	*Zm00001d005998*	GCAGCTCCTGATCGAGATTC	GCGACCCTATCTGGTCGTAG	2	194954891..194956278
CCR	*Zm00001d032152*	GCTGCTCGAGAAGGGATACAC	CGGGTCATCTGCTGCCAT	1	214573400..214579274
CAD	*Zm00001d002346*	TGCAGATCTCTGCCTTCTCTTT	GCGTTCCATGGCCTCATT	2	10649171..10653306
POD	*Zm00001d009138*	AAGCGCGTGGAAGACCAA	TGCCGTGTGAAGCTGAAATAA	8	38826820..38828682
POD	*Zm00001d053554*	GCCCTCTTCGCCTCAGACTT	TCAGCTGCTGTTGACCACCC	4	233852931..233854306
MYB	*Zm00001d014029*	AACTACCTCCACCCGGACAT	GTGCCACACGTTCTTGATCTC	5	29347095..29349160
MYB	*Zm00001d040621*	GGCTGAGGCTGGTTTTGCT	GTTCGACCGGGCAAGTGA	3	54361983..54365788
MYB	*Zm00001d008808*	TAGGAAGTGCTCTAGCTGTGGG	TGAAGCACCTCTTCATGGCTA	8	21238420..21240608
MYB	*Zm00001d009435*	GGGGAACAGGTGGTCGCA	GCGGCTTGCTCTGGTCGT	8	64426126..64427468
MYB	*Zm00001d038288*	GCTGTTTCTGGAGGGGCTT	GTCGTGGATGCTCTTGCG	6	153438814..153440041
MYB	*Zm00001d044117*	AGTGGACGGCGAAGCAGAA	GTAGCGCCTGACCTCGTCCGCC	3	219796585..219798521
NAC	*Zm00001d003626*	TGTTTGTGCTGACGAGACGG	CGTCGAATCAAACACTCGGCATATC	2	50875214..50876868
NAC	*Zm00001d006053*	GCTTCAGGTTCCACCCGACT	CGTTCGCACGCCTGTTGGGTA	2	196582113..196584517
NAC	*Zm00001d020982*	TGGTGGTGGAGGGGGGAAGTCG	GACCGGTGAAGATTTCCTTATC	7	138832013..138836335
NAC	*Zm00001d022517*	CGCTGGACAGCCGCTTCTCCTG	CTGCTACTGCCGCGGAAGAT	7	179273894..179275680
WRKY	*Zm00001d008794*	GGAGGAACAAGAACAACGCA	CGAGGTCGTCGGGGTCGTCGGT	8	20545292..20547558
WRKY	*Zm00001d012505*	ACGAGATCGCCTACTACGGG	TCCAGGAACTCCATTACCGGATCAG	8	175577029..175578804
PEX14	*Zm00001d011119*	GCAGGTCAACAGCCAGAATCA	AGCACCAAAACCAAGCAAGA	8	39412827..139416787
PXMP2	*Zm00001d052410*	CCATTTTCATTGGCGTCTTTA	CAAGGGCTACAAAATTAGCACC	4	189313573..189318465
MPV17	*Zm00001d011761*	CGGCTATGGGTGATTCCTTT	GGTTTCATTTGCTCATTGTGGT	8	160949609..160951534
ACOX	*Zm00001d045606*	GTCATCCACAGTCCAACTCTCAC	CACTACCGAACTTTCCACCAAT	9	28565344..28570133
ACAA1	*Zm00001d049882*	AGGCAAGAGCAAGATCAGACC	ATCCACTCCAACAGCGGCA	4	50211783..50214016
CAT	*Zm00001d027511*	CGCACACCACAACAACCACT	ACCGCTCTTGCCTTGCTG	1	7144389..7146665
SOD	*Zm00001d031908*	GCATAGCCGAGGCAACCAT	CCCCCTTTCCCCAAATCAT	1	206405747..206408926
MVK	*Zm00001d054008*	CTCCGCCTTCCTTTTTCTCT	CAACACCTCCCAACCCTCA	4	245047670..245055426

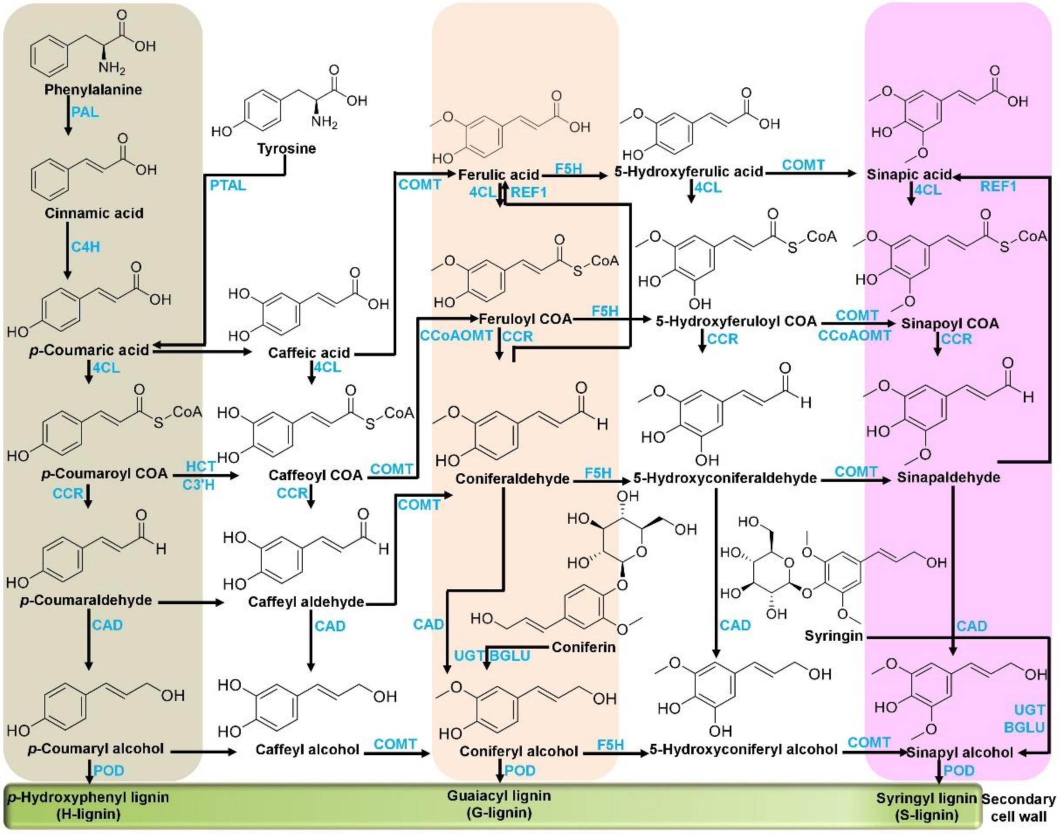

Figure S1. Phenylpropanoid pathway leading to lignin biosynthesis was showed in maize (phenylalanine ammonia-lyase [PAL], phenylalanine/tyrosine ammonia-lyase [PTAL], trans-cinnamate 4-monooxygenase [C4H], caffeoyl-CoA O-methyltransferase [CCoAOMT], caffeic acid O-methyltransferase [COMT], coniferyl-aldehyde dehydrogenase [REF1], 4-coumarate-CoA ligase [4CL], 5-O-(4-coumaroyl)-D-quinate 3'-monooxygenase [C3'H], ferulate-5-hydroxylase [F5H], shikimate O-hydroxycinnamoyltransferase [HCT], cinnamoyl-CoA reductase [CCR], cinnamyl-alcohol dehydrogenase [CAD], peroxidase [POD], laccase [LAC], beta-glucosidase [BGLU], and UDP-glucosyltransferase [UGT]).

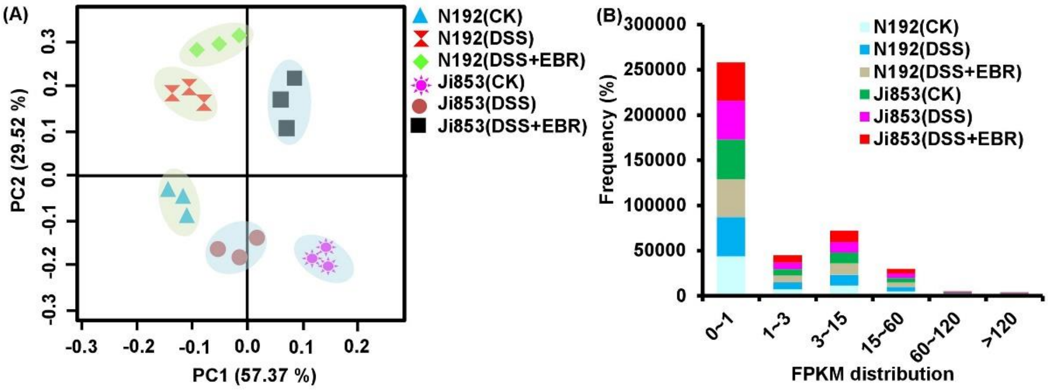

Figure S2. The principal component analysis (PCA) (A) and FPKM values distribution of corresponding genes (B) in N192 and Ji853 mesocotyls under 3 cm sowing depth (CK), 20 cm sowing depth (DSS), 4.16×10^{-3} M 24-epibrassinolide (EBR) induction at 20 cm sowing depth (DSS+EBR) conditions by RNA-sequencing (RNA-Seq) data.

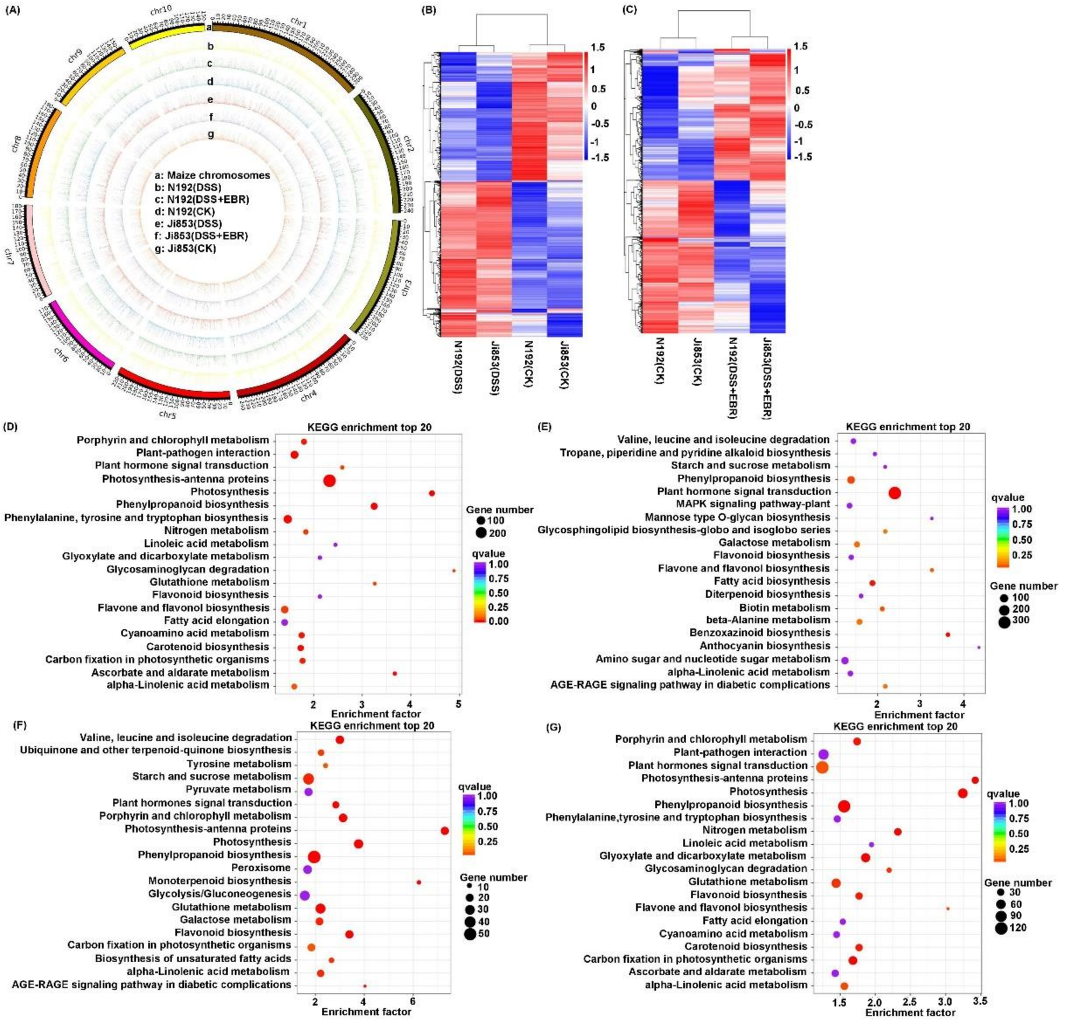

Figure S3. Distribution and expression profiles of differentially expressed genes (DEGs) and top 20 KEGG enrichment analysis in N192 and Ji853 mesocotyls under CK, DSS, and DSS+EBR conditions. Distribution of DEGs in N192 and Ji853 mesocotyl under three conditions (A). Based on the log2 fold-change value of different comparisons, DEGs hierarchical clustering performed for three conditions to obtain six clusters (B and C). Top 20 KEGG pathways enriched by DEGs in N192 and Ji853 mesocotyls under DSS conditions, respectively (D and E), and which under DSS+EBR conditions, respectively (F and G), respectively.

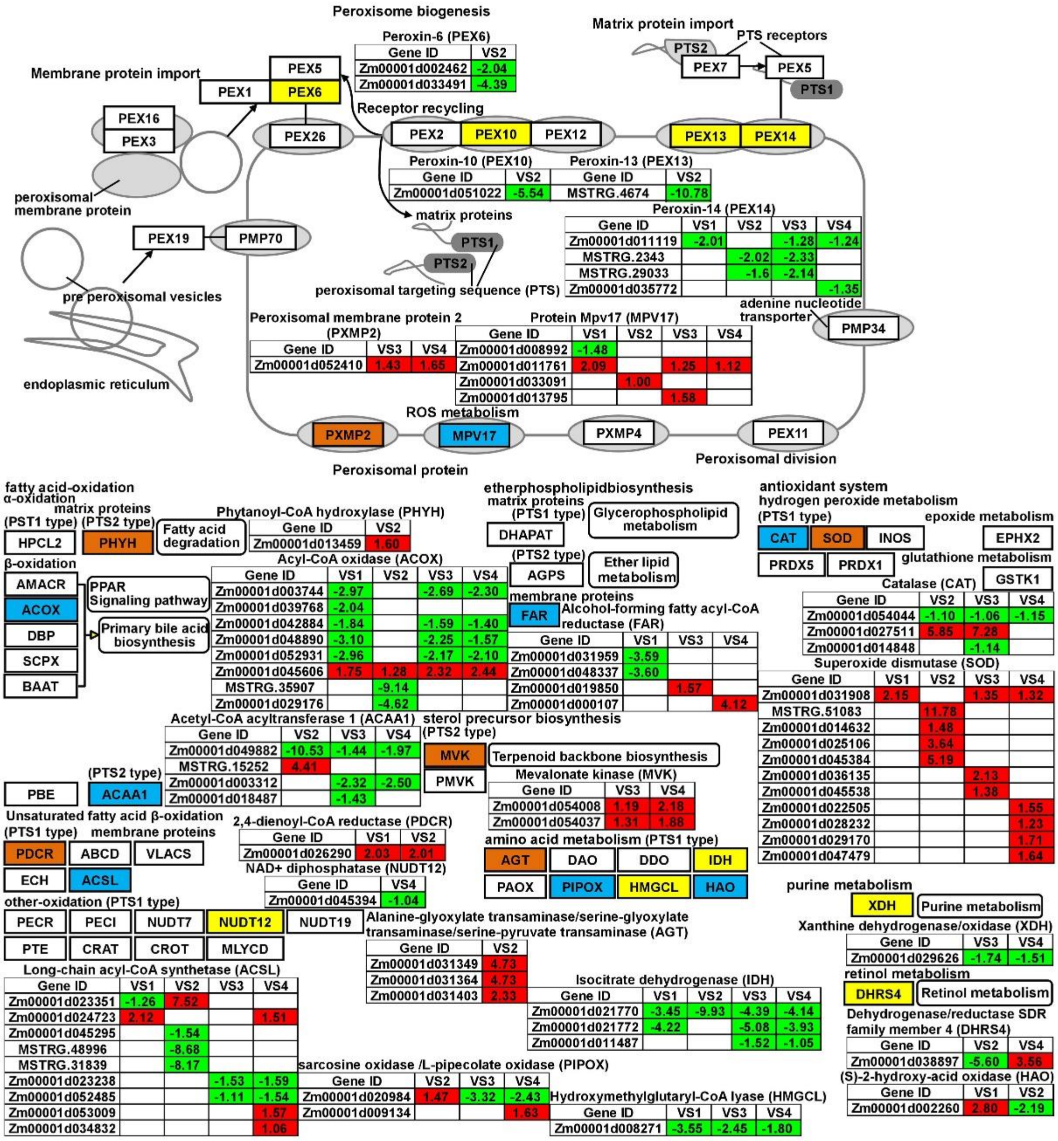

Figure S4. DEGs involved in peroxisome biogenesis that controlled lignin biosynthesis by regulating H_2O_2 level in N192 and Ji853 mesocotyls under CK, DSS, and DSS+EBR conditions.

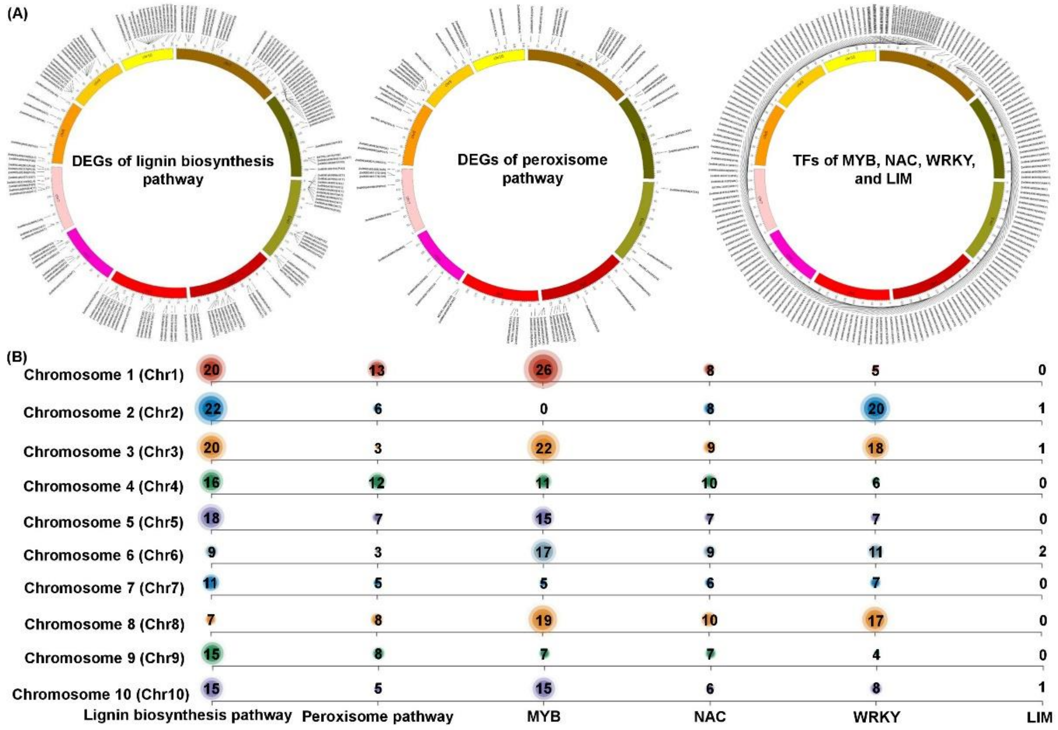

Figure S5. Genomic distributions of corresponding identified DEGs/transcription factors (TFs) on ten maize chromosomes. DEGs of lignin biosynthesis pathway, peroxisome pathway, and TFs including MYB, NAC, WRKY, and LIM distributions map (A). Bubble diagram of number for detected DEGs on each maize chromosome (B).

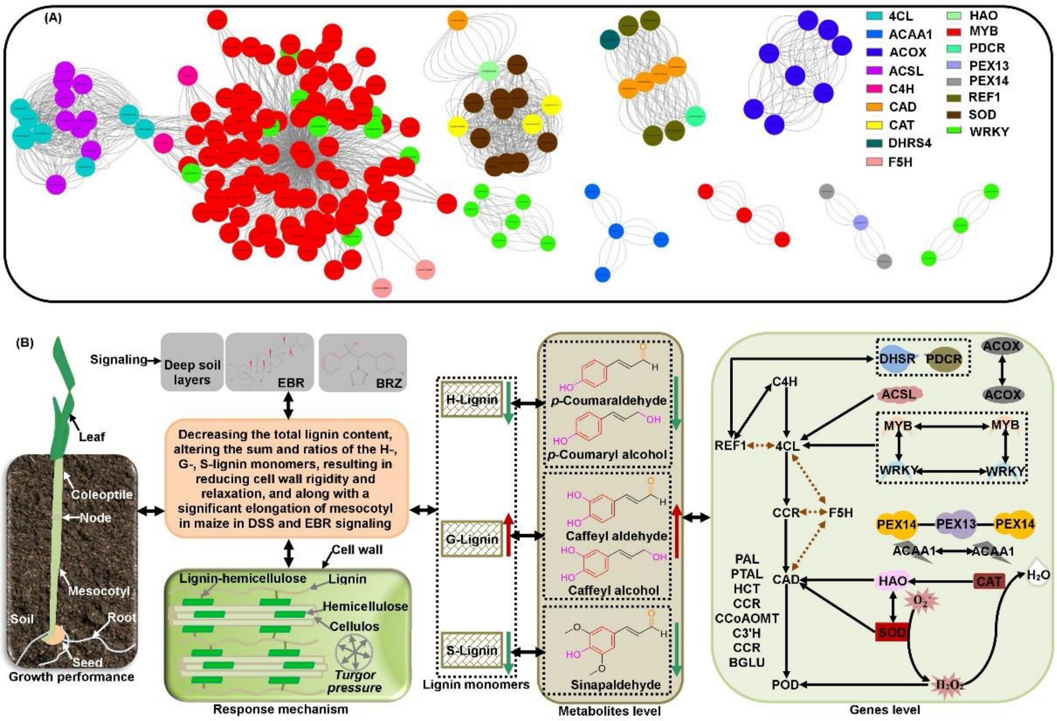

Figure S6. Interaction networks of detected DEGs and molecular network underlying the DSS and EBR signaling response of maize mesocotyl elongation. Detected DEGs of lignin biosynthesis pathway, peroxisome pathway, and corresponding TFs interactome network mapping in maize mesocotyl in DSS and EBR signaling (A). The schematic molecular model of maize mesocotyl elongation in DSS and EBR signaling (B).
